# The Importance of Early Endovascular Intervention and Guideline-Based Cardiac Rehabilitation When Managing Type-B Aortic Dissection

**DOI:** 10.7759/cureus.85206

**Published:** 2025-06-01

**Authors:** Daniel Chadda, Ramtin Khanipour, Mohammad Z Rehman, Charles Boadu, Nader Chadda

**Affiliations:** 1 Cardiology, George Washington University, Washington, D.C., USA; 2 Internal Medicine and Cardiology, HCA Florida Bayonet Point Hospital, University of South Florida, Hudson, USA; 3 Internal Medicine, Medical Center of Trinity, Trinity, USA; 4 Interventional Cardiology, Advanced Heart and Vascular, Hudson, USA

**Keywords:** acute aortic syndrome, aortic aneurysm, aortic dissection complications, aortic rupture, cardiac rehabilitation, endovascular intervention, hypertension management, thoracic endovascular aortic repair, type-b aortic dissection

## Abstract

Most type-B aortic dissections are managed medically, except in certain situations such as descending aortic rupture, malperfusion, hypertension refractory to medical therapy (β-blockers and dihydropyridine calcium channel blockers), aneurysmal dilation, expansion, rupture, or impending rupture, where surgical intervention or an endovascular procedure is recommended as the primary method of treatment.

However, there are scenarios where a type-B aortic dissection meets the criteria for surgical or endovascular intervention, but the patient is a poor surgical candidate due to the presence of numerous comorbidities (e.g., advanced age, hemodynamic instability, or the absence of a sufficient “landing zone” for intervention).

In the following case report, we describe a 76-year-old female who presents with type-B aortic dissection extending to the distal left iliac artery. During her hospitalization, the patient was managed medically. Unfortunately, on day five of hospitalization, the patient developed sudden-onset chest pain followed by cardiac arrest shortly after her cardiac rehabilitation session. Considering how extensive her type-B aortic dissection was, it is hypothesized that due to physical exertion, there was an acute-onset retrograde expansion of the flap leading to sudden cardiac death. This emphasizes the importance of early surgical intervention as well as having a strict guideline regarding the level of physical exertion that such patients can undergo.

## Introduction

Aortic dissection is the tearing of the tunica intima, allowing blood to penetrate the wall and causing the dissection (separation) of the tunica intima from the tunica media. There are two classification systems that are used to categorize aortic dissections: 1) the Stanford system (more common) and 2) the DeBakey classification. According to the Stanford system, aortic dissection can be subcategorized into type-A (involving the ascending aorta and proximal to the brachiocephalic artery) and type-B (involving the descending aorta distal to the left subclavian artery). Risk factors associated with aortic dissection consist of long-standing hypertension, genetic disorder (e.g., Marfan syndrome, Ehlers-Danlos syndrome, bicuspid aortic valve, and coarctation of the aorta), vasculitis (e.g., syphilis), trauma, and cocaine use [1].

Aortic dissection usually manifests with sudden, severe tearing chest pain radiating to the back between the shoulder blades. Symptoms could mimic myocardial infarction, but in hemodynamically stable patients, the diagnosis can be confirmed by using CT angiography of the chest. On the other hand, in the case of hemodynamically unstable patients or patients with kidney disorders or contrast allergies, the preferred diagnostic test is the transesophageal echocardiogram (TEE). The treatment for type-A aortic dissection is a surgical emergency, whereas type-B is preferentially managed medically with blood pressure-lowering medications (e.g., beta-blockers), except when there are complications such as descending aortic rupture, malperfusion, hypertension refractory to medical therapy, aneurysmal dilation (>55 mm), expansion, rupture, or impending rupture [2-4]. These complications are an indication for urgent surgery or thoracic endovascular aortic repair (TEVAR).

In this case report, we focus on elaborating on the urgency of a surgical or interventional approach in patients with complicated aortic dissection as well as the role that physical therapists play in allowing for a safe discharge from the hospital.

## Case presentation

A 76-year-old female with a past medical history of hypertension (HTN), hyperlipidemia, and chronic back pain presented to the emergency room with a sudden syncopal episode, retrosternal chest pain radiating to the back, and left lower extremity weakness and numbness. CT angiography of the chest revealed an aortic dissection. The patient was airlifted to our hospital for further evaluation by cardiothoracic surgery (CTS). She arrived on a nicardipine drip, hemodynamically stable, and neurologically intact with no focal deficits on initial assessment. She described the chest pain as dull intermittent pain, 9/10 in intensity, radiating to the back, with no aggravating or relieving factors. During a physical examination in the emergency department, she was awake and alert. Her heart rate was 71 beats per minute (bpm), and her blood pressure was 129/62 mmHg. The abdomen was soft with moderate tenderness in the epigastric area. The patient was moving all extremities, and distal pulses were intact. The patient was placed on an esmolol drip and admitted to the intensive care unit (ICU) for closer monitoring. 

**Figure 1:**
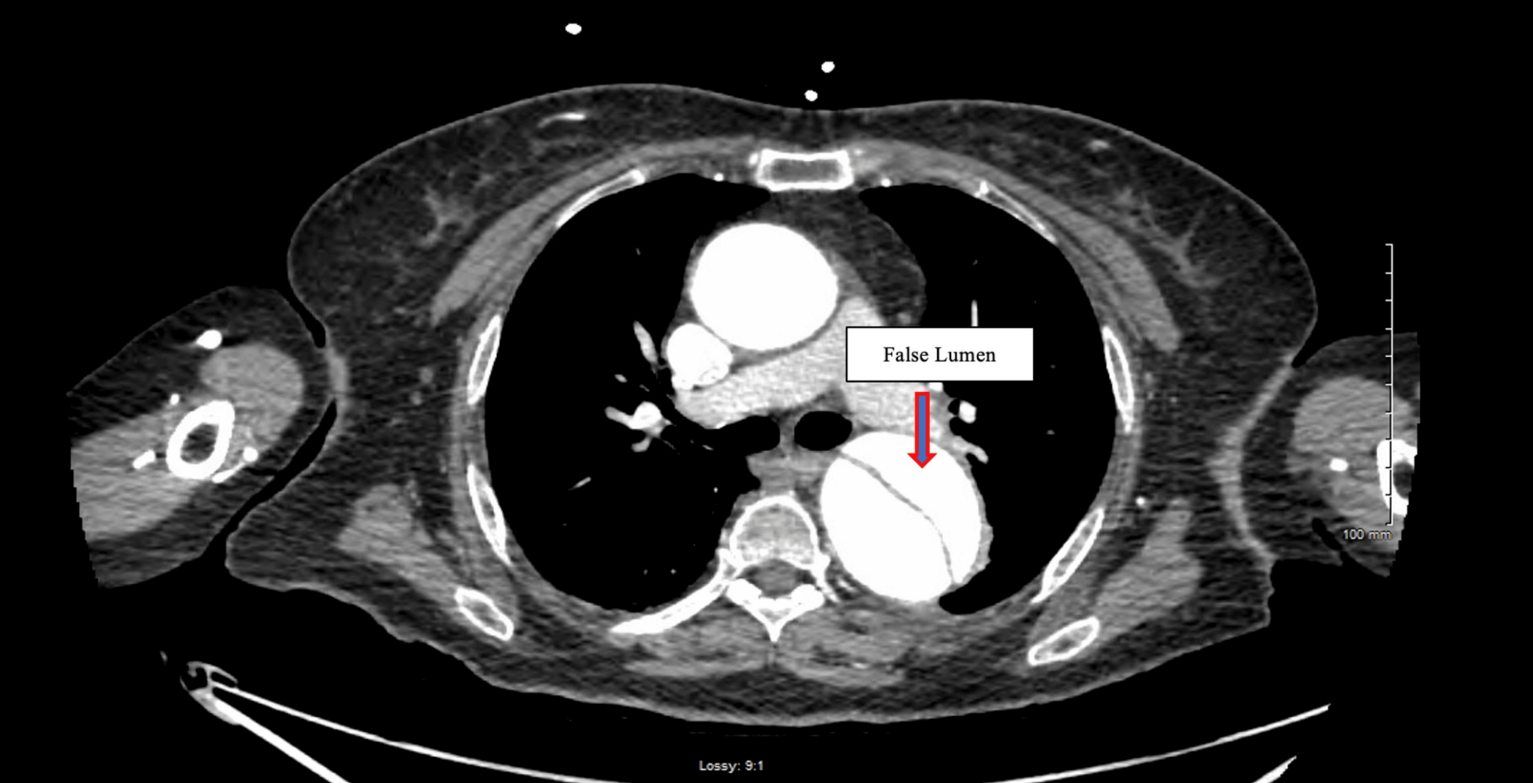
Start of type-B aortic dissection at the level of proximal descending aorta

**Figure 2:**
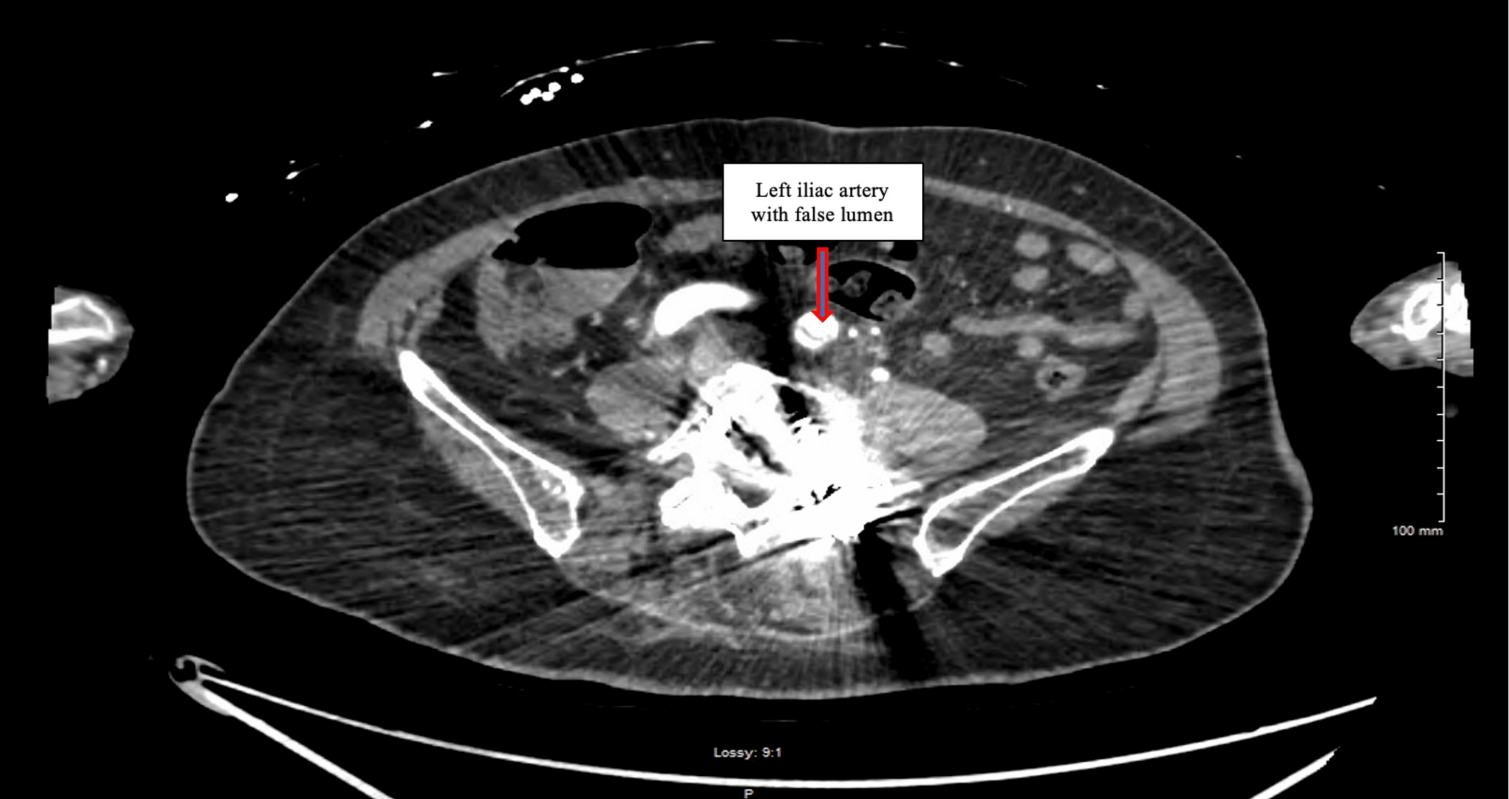
Continuation of aortic dissection at the level of left iliac artery

**Figure 3:**
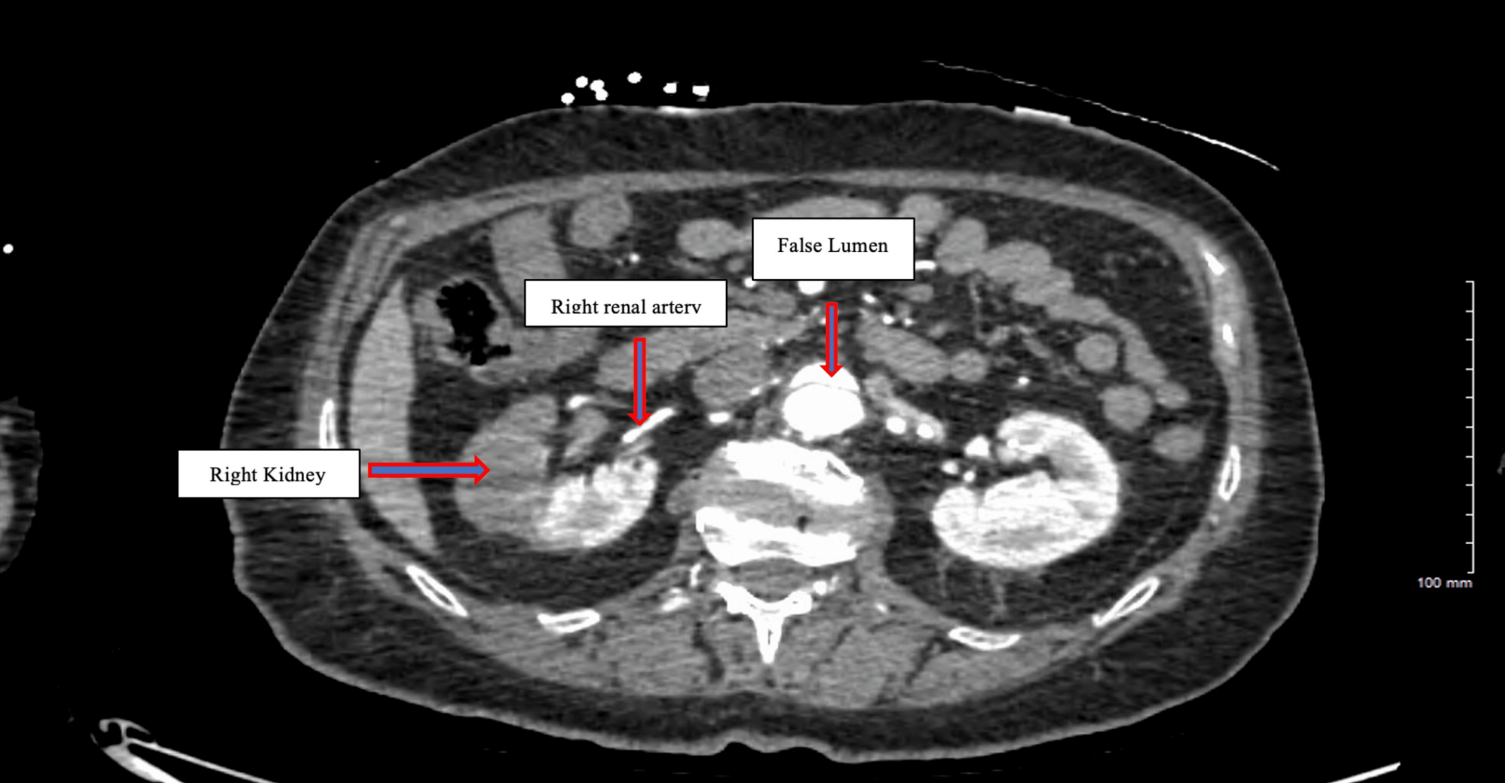
Extension of type-B aortic dissection at the level of kidneys with right kidney being supplied by false lumen and left kidney being supplied by true lumen

Aortic computed tomographic angiography (CTA) with distal runoff revealed a type-B aortic dissection starting from the proximal descending thoracic aorta (Figure 1) and extending to the distal left iliac artery (Figure 2). The true lumen supplied the left renal artery, while the right renal artery arose from the false lumen (Figure 3). Additionally, ascending and proximal thoracic descending aortic aneurysms were noted on CTA.

The patient remained in the ICU throughout the hospital course. The cardiovascular team unanimously recommended medical management, initially with esmolol and nicardipine drips, which were later transitioned to p.m. labetalol and amlodipine. Repeat imaging (3 days later) showed a 4 mm increase in descending aortic dilation and diminished flow in the false lumen. Initial serum creatinine (Cr) on admission was 1.0, and on day 3 it spiked to 1.4. During this same period, the Glomerular Filtration Rate (GFR) decreased from 58.4 ml/min to 39.0 ml/min. CTS at this time classified the patient as a “high risk,” given her advanced age and lack of a landing zone for surgical intervention. The interventional cardiologist on the case stated that the patient is not an appropriate candidate for TEVAR since the dissection extends down to the left common iliac artery, and it would be technically challenging to achieve an optimal result. The patient continued treatment with medical management. 

Key laboratory findings are summarized in Table 1.

**Table 1:** Lab Results BUN: blood urea nitrogen; Est GFR (CKD-EPI 2021): estimated glomerular filtration rate (Chronic Kidney Disease Epidemiology Collaboration 2021); AST: aspartate aminotransferase; ALT: alanine aminotransferase; Hgb: hemoglobin; Hct: hematocrit; MCV: mean corpuscular volume; MCH: mean corpuscular hemoglobin; MCHC: mean corpuscular hemoglobin concentration; RDW: red cell distribution width; Plt: platelet; PT: prothrombin time; INR: international normalized ratio

	Nov 21, 2023, 03:42	Nov 20, 2023, 06:11	Nov 19, 2023, 04:31	Nov 18, 2023, 04:00	Nov 17, 2023, 2:02	Nov 16, 2023, 14:13	Nov 16, 2023, 17:15	Nov 16, 2023, 00:00	Reference Range
Sodium	132	132	132	136	136	137	137	137	135-145 mmol/L
Potassium	3.8	4.0	4.1	4.6	4.4	4.2	4.3	3.4	3.5-5.1 mmol/L
Chloride	101	101	102	106	107	106	106	105	98-107 mmol/L
Carbon Dioxide	23	21	23	23	24	24	23	26	22-29 mmol/L
Anion Gap	8	10	7	7	5	7	8	6	3-11 mmol/L
BUN	27	30	25	24	20	15	13	12	7-20 mmol/L
Creatinine	1.30	1.20	1.10	1.20	1.40 H	1.2	1.0	1.0	0.6-1.3 mg/dL
Est GFR (CKD-EPI 2021)	42.6	46.9	52.1	46.9	39	46.9	58.4	58.4	>60 mL/min/1.73m^2
BUN/Creatinine Ratio	20	25H	22	20	14	12	13	12	10:1-20:1
Glucose	104	100	121	131	118	136	141	182	70-99 mg/dL
Calcium	8.4	8.6	8.3	8.1	7.8	8.1	8.7	8.6	8.5-10.5 mg/dL
Phosphorous	3.9	3.3	2.7	3.1	4.3				2.5-4.5 mg/dL
Magnesium	1.9	2.2	2.0	2.1	1.9				1.6-2.6 mg/dL
Total Bilirubin	0.4	0.6	0.6	0.5	0.8		0.6	0.6	0.1-1.2 mg/dL
AST	15	16	20	33	22		15	18	10-40 U/L
ALT	14	17	14	16	15		14	15	7-56 U/L
Alkaline Phosphatase	41	41	41	35	37		49	54	44-147 U/L
Total Protein	5.6	5.9	6.0	5.5	5.7		6.4	6.9	6.0-8.3 g/dL
WBC	6.3	6.3	8.3	9.1	9.9		8.6	9.3	4.0-11.0 x10^3/uL
RBC	3.05	3.16	3.18	3.04	3.1		3.63	3.83	4.2-5.9 x10^6/uL
Hgb	9.4	9.8	10	9.4	9.7		11.3	11.8	12.0-15.5 g/dL
Hct	27.8	28.7	28.9	28.9	29.9		34.6	35.1	34.9-44.5%
MCV	91.1	90.8	90.9	95.1	96.5		95.3	91.6	80-100 fL
MCH	30.8	31	31.4	30.9	31.3		31.1	30.8	27-33 pg
MCHC	33.8	34.1	34.6	32.5	32.4		32.7	33.6	32-36 g/dL
RDW	12.6	12.4	12.7	12.9	13		12.6	12.5	11.5-14.5%
Plt Count	205	207	171	143	147		167	170	150-450 x10^3/uL
PT								12.2	11-13.5 seconds
INR								1.1	0.8-1.1

On day five, after working with the physical therapist, the patient suddenly developed a sense of impending doom, sat up on her bed, and started to become lethargic and unresponsive. She started to become bradycardic, hypotensive, and cold to the touch, with no palpable distal pulses. Bedside telemetry showed ST elevations, and since the patient was a DNR (Do Not Resuscitate), CPR was not initiated. The patient eventually went into asystole and subsequently passed away peacefully.

## Discussion

Acute aortic dissection is a critical condition with an approximate mortality rate of 1-2% per hour in the first 24 hours and up to 80% within the first two weeks from the onset if not promptly treated [5]. In most patients with type-B aortic dissection, medical therapy including analgesia, antihypertensive drugs (beta-blockers), and bed rest is performed [2, 6]. However, complicated type-B aortic dissection, such as descending aortic rupture, malperfusion, hypertension refractory to medical therapy, aneurysmal dilation (>55 mm), expansion, rupture, or impending rupture, is an indication for urgent surgery or TEVAR [2-4]. The aim of surgical repair in patients with type-B aortic dissection is to resect the primary entry tear and replace the dissected descending aorta, which increases blood flow to the true lumen and improves organ ischemia [2]. However, the role of early TEVAR for uncomplicated type-B aortic dissection is controversial [3]. The current guideline indicates that in the case of acute type-B aortic dissection with >4 cm aortic diameter and high surgical risks, then TEVAR would be the preferred treatment approach [7].

About 30-42% of acute type-B aortic dissections are complicated by hemodynamic instability and peripheral vascular ischemia requiring surgical or TEVAR interventions. Considering the high mortality rate, prolonged hospitalization, and associated cost with open surgical intervention, TEVAR is a minimally invasive procedure that is used as an alternative for treating complicated type-B aortic dissection in patients who are at high surgical risk, such as our patient. By allowing for the closure of the main entry in dissection, this minimally invasive endovascular procedurecan relieve the wall stress, leading to shrinkage of the false lumen, allowing for proper remodeling of the dissection, and resolution of malperfusion, which is commonly associated with complicated type-B aortic dissection. Studies have illustrated that patients have a higher level of satisfaction and improved survival rate in those who receive early endovascular repair as opposed to those who solely rely on medical therapy [5].

Cardiac rehab is a vital component of the postoperative care of patients with acute aortic dissection. Early and appropriately intensified rehab can significantly enhance recovery and long-term outcomes. Usually, the first 14 days after symptom onset are referred to as “acute” aortic dissection, which has the highest rates of mortality and morbidity. With the completion of this acute phase, blood pressure tends to stabilize, symptoms become less severe, and at that point, aortic dissection is considered to be “chronic” [1]. Cardiac rehab during the acute phase should be carefully planned, require supervision, and have an individualized approach to mitigate risks and maximize benefits. Strategies for implementing cardiac rehabilitation in patients with acute aortic dissection are not well established, with little evidence to stratify prudent and effective guidelines [8].

We recommend that the intensity of cardiac rehabilitation should be increased gradually, based on the patient's recovery and stability. The initial phase should focus on gentle, low-intensity activities, breathing exercises, and light walking. As the patient’s condition improves, the intensity can be increased in a structured manner. Eventually, the patient can engage in moderate-intensity exercises, under supervision, tailored to their specific needs and response to therapy. Continuous monitoring/recording of blood pressure and heart rate before and during rehab sessions (the physical therapist failed to complete this task for our patient) to detect any signs of distress, as well as clear communication between the physical therapist and cardiologist/CTS team, is critical to ensure safe and coordinated care.

One possible explanation for sudden death** **is that the patient could have developed acute-onset massive pulmonary embolus (PE) because of immobility and lack of deep vein thrombosis** **prophylaxis, secondary to active aortic dissection, resulting in sudden cardiac arrest. However, considering the size of the dissection, the likely scenario in our case was that the patient overexerted herself during cardiac rehabilitation, leading to the retrograde expansion of the flap with extension into either the carotids and coronaries or rupture of the aneurysm, leading to hemorrhagic shock. This case advocates for early surgical/endovascular interventions when feasible and the pressing need for guideline-directed cardiac rehabilitation in acute aortic dissection.

## Conclusions

Management of type-B aortic dissection typically involves medical therapy, but surgical/endovascular interventions are warranted in specific situations where complications arise or medical therapy fails. Additionally, cardiac rehabilitation should commence early but must be cautiously managed, starting with low-intensity activities and gradually increasing based on the patient’s tolerance and recovery. Continuous monitoring and a personalized rehabilitation plan are essential for optimal recovery.
